# Alterations in Neural Control of Constant Isometric Contraction with the Size of Error Feedback

**DOI:** 10.1371/journal.pone.0170824

**Published:** 2017-01-26

**Authors:** Ing-Shiou Hwang, Yen-Ting Lin, Wei-Min Huang, Zong-Ru Yang, Chia-Ling Hu, Yi-Ching Chen

**Affiliations:** 1 Institute of Allied Health Sciences, College of Medicine, National Cheng Kung University, Tainan City, Taiwan; 2 Department of Physical Therapy, College of Medicine, National Cheng Kung University, Tainan City, Taiwan; 3 Physical Education Office, Asian University, Taichung City, Taiwan; 4 Department of Management Information System, National Chung Cheng University, Chia-Yi, Taiwan; 5 School of Physical Therapy, College of Medical Science and Technology, Chung Shan Medical University, Taichung City, Taiwan; 6 Physical Therapy Room, Chung Shan Medical University Hospital, Taichung City, Taiwan; University of Illinois at Urbana-Champaign, UNITED STATES

## Abstract

Discharge patterns from a population of motor units (MUs) were estimated with multi-channel surface electromyogram and signal processing techniques to investigate parametric differences in low-frequency force fluctuations, MU discharges, and force-discharge relation during static force-tracking with varying sizes of execution error presented via visual feedback. Fourteen healthy adults produced isometric force at 10% of maximal voluntary contraction through index abduction under three visual conditions that scaled execution errors with different amplification factors. Error-augmentation feedback that used a high amplification factor (HAF) to potentiate visualized error size resulted in higher sample entropy, mean frequency, ratio of high-frequency components, and spectral dispersion of force fluctuations than those of error-reducing feedback using a low amplification factor (LAF). In the HAF condition, MUs with relatively high recruitment thresholds in the dorsal interosseous muscle exhibited a larger coefficient of variation for inter-spike intervals and a greater spectral peak of the pooled MU coherence at 13–35 Hz than did those in the LAF condition. Manipulation of the size of error feedback altered the force-discharge relation, which was characterized with non-linear approaches such as mutual information and cross sample entropy. The association of force fluctuations and global discharge trace decreased with increasing error amplification factor. Our findings provide direct neurophysiological evidence that favors motor training using error-augmentation feedback. Amplification of the visualized error size of visual feedback could enrich force gradation strategies during static force-tracking, pertaining to selective increases in the discharge variability of higher-threshold MUs that receive greater common oscillatory inputs in the β-band.

## Introduction

Force steadiness is a useful paradigm for investigating fine motor control and perceptuo-motor variability [1, 2]. During constant isometric contraction, the smoothness of a force trajectory is undermined by numerous intermittent drifts of the force output away from the target force, known as force fluctuations. Accumulating evidence has convincingly shown that force variability, particularly for those low-frequency force fluctuations under 4 Hz, are related to visuomotor processes [3, 4, 5] and movement corrections [6, 7]. The amount of visual spatial information can influence the properties of force fluctuations. For static isometric contraction at very low exertion levels (2%-10% maximal voluntary contraction), the majority of previous studies reported that a high spatial resolution of the visual display (or a smaller change in force occupying a greater number of pixels) produced superior force steadiness for the young adults, supported by reduction in the size of force fluctuations [8, 9, 10]. Although motor unit discharge is a major determinant of force fluctuations [11, 12], the mean and coefficient of variation of the inter-spike interval of motor units of young adults are unexpectedly not tuned to visual spatial information [3, 13], except that Laine et al [14] reported that all spectral bands of the common oscillatory input to motor units varied with the spatial resolution of a visual display. In addition to experimental contexts, some of the inconsistency in the findings on the visual impact on force fluctuations and motor unit discharge was due to the failure of previous studies to specify the roles of high-threshold motor units. On account of lower and more variant discharge rates, high-threshold motor units with a larger twitch force are liable to produce a greater size of force fluctuations [11, 15].

Execution errors during continuous adjustments of the planned motor goal are critical to improving task quality [16]. Error detection relies predominantly on visual feedback to reduce perception uncertainty from the proprioceptive and haptic inputs. Distorted visual feedback could degrade task performance before the participants develop a new calibration process to override the biased perception-action link. Intriguingly, error-augmentation feedback, a particular form of distorted visual feedback, does not exert a negative impact on task success. For point-to-point movements with visual rotation, previous kinematic studies showed that augmenting trajectory errors by adding a fixed bias to the original error could expedite motor adaptations to novel task constraints [17, 18, 19]. Although spatiotemporal tuning of force performance of central origins is known to selectively involve in striatal—frontal circuit and parietal—frontal circuit [20, 21], yet the neural mechanisms underlying variations in force performance with the error size are not fully lucid especially in the aspects of motor unit physiology. In real practice, the augmentation of execution errors via visual display is considered to be effective for facilitating motor recovery of patients with neurological disorders [19, 22].

If error augmentation could increase control of force steadiness, low-frequency force fluctuations and motor unit behaviors should show characteristic changes in favor of fine force-tuning processes, and vice versa. Employing multi-electrode surface electromyography technology and decomposition procedures, this study aimed to examine how the size of visualized execution error alter motor unit discharge and force-discharge relation during force-tracking at a fixed exertion level. Our main hypotheses were that 1) force steadiness, high frequency components, spectral dispersion, and complexity of force fluctuations would augment with increasing error amplification factor that virtually multiplied the size of execution error, and vice versa, 2) the inter-spike intervals of MUs (mean and variability) and common oscillatory drives to MUs would increase with larger error amplification factor, particularly for motor units with relatively high recruitment thresholds, and 3) the force-discharge relation would be complicated by increasing error amplification factor.

## Materials and Methods

The participants were 14 healthy adults (6 males and 8 females; mean age: 25.87 ± 1.19 years, range: 21–35 years old) from a university campus or the local community. All were self-reported as being right-handed, and none had symptoms or signs of neuromuscular diseases. The research project was approved by an authorized institutional human research review board (IRB) at the University Hospital of the National Cheng Kung University, Taiwan. All of the participants signed an informed consent before the experiment, conforming to the Declaration of Helsinki.

### Experimental Procedures

The participants completed a unilateral static force task (isometric index abduction) under three error feedback conditions (low amplification factor (LAF), normal amplification factor (NAF), and high amplification factor (HAF)) at a low force level (10% maximal voluntary contraction (MVC)). A low force level was chosen to prevent neuromuscular fatigue during the multiple trials in this study. Each condition contained 3 experimental trials interleaved with 3-minute pauses. Experimental trials were executed in a randomized order. The participant was seated with his/her palm and forearm of the left hand firmly fixed within a thermoplastic splint on the table. We considered the non-dominant limb for the force-tracking task, because it would be more novel to the non-dominant limb as compared with the dominant limbs [23]. The index finger was held slightly abducted (5 degrees of abduction), and its abduction force was measured using a force transducer (Model: MB-100, Interface Inc., Scottsdale, AZ, USA). With verbal encouragement, the maximal voluntary contraction (MVC) of the first dorsal interosseus (FDI) was determined by three contraction trials of index abduction with maximal effort of 5 s separated by 3 min pauses. The average of the highest force value produced in each trial was the respective MVC for each individual.

**Fig 1:**
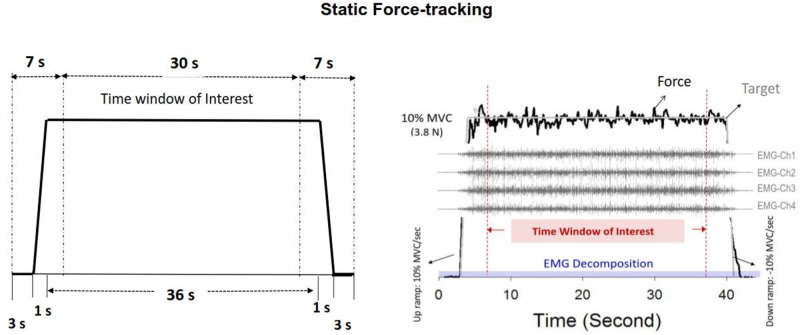
Schematic diagrams of task protocol and recording of physiological data. The force task required the participant to couple force output with the target signal (left plot) under three different feedback conditions. EMG decomposition analysis was performed throughout the 44 s data collection period (right plot), whereas only behavior and discharge variables from the 7^th^ to the 37^th^ seconds were considered, as data in the time window of interest were considered relatively stable.

The experiment commenced after a rest period of 20 minutes. All the participants were given three practice trials in each feedback condition (LAF, NAF, and HAF). In the practice trials and the following experimental trials, the participants were instructed to produce an isometric force by pushing their index finger against the force transducer and to match the force produced to the target force line (10% MVC) displayed on the monitor (1024×768 pixels). After a latent period of 3 seconds, the participants were given 1 second to reach the target force and steadily maintained that force for 36 seconds under visual guidance (Fig 1). Then the force output returned back to the resting state in 1 second, followed by another 3-second latent period. An experimental trial took 44 seconds to complete. The time window of interest was denoted as the 7th to 37th seconds of the experimental trial.

**Fig 2:**
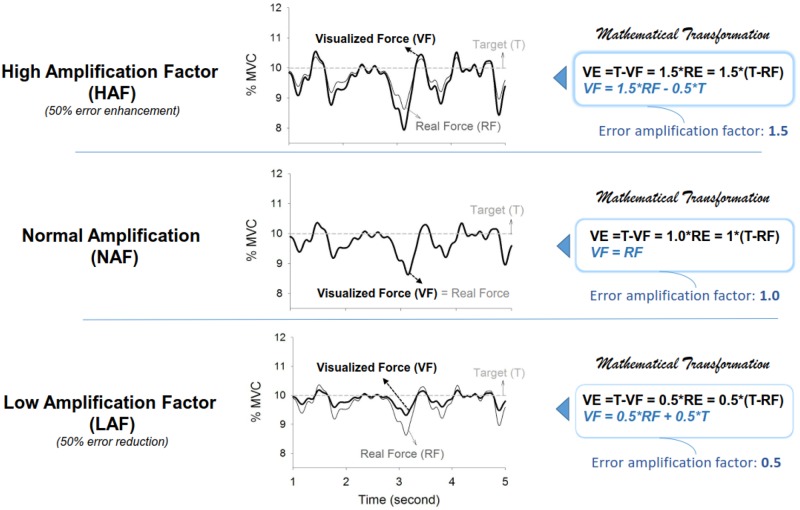
Illustration of modulation of error feedback gain. Following mathematical transformation, the visualized force outputs (black bold line) represent an on-line force trajectory that augments and reduces by 50% the original real tracking error (grey thin line) in the high amplification (HAF) and low amplification (LAF) conditions, respectively. In the normal amplification (NAF) condition, visualized force output is equal to real force output, such that the participant perceives the real tracking error during force-tracking. (VF: visualized force; VE: visualized error; RF: real force; RE: real error; T: target signal)

The force feedback of the three feedback conditions was mathematically transformed in three different ways before it was displayed on the monitor (Fig 2) such that the participants could visualize different degrees of mismatches between the same actual force output and the target signal. In the low amplification (LAF) condition, the force output displayed on the monitor (visualized force, VF) was equal to the sum of half of the real force (RF) plus half of the target signal (T)(VF = 0.5*RF+0.5*T). The size of the visualized tracking error (VE) was reduced so that the participant saw half of the real error (RE) of the static force-tracking task (VE = 0.5*RE). In the normal amplification (NAF) condition, the participant was provided with real error (VE = RE), as the visualized force output was identical to real force output. In the high amplification (HAF) condition, the force output displayed on the monitor (VF) was transformed with VF = 1.5*RF-0.5*T. The size of the perceived tracking error was augmented by 50% during the static force-tracking task in the HAF condition (VE = 1.5*RE). To accentuate the differences, the spatial gain to the display target signal and the force output was identical for all experimental conditions, with the visual resolution set at 15 pixels per 1% MVC. The representation of visual gain with pixels per 1% MVC rather than pixels to per Newton was to minimize potential strength effect across the participants on visual display. The inter-trial interval of rest was 3 minutes to minimize a potential fatigue effect. The force signal was sampled at 1 kHz by an analog-to-digital converter with 16-bit resolution (DAQCard-6024E; National Instruments Inc., Austin, TX, USA), controlled by a custom program on a LabVIEW platform (LabVIEW v.8.5, National Instruments Inc., Austin, TX, USA).

### Electromyographic Recordings

Synchronized with the force signal, multi-electrode surface electromyography (EMG)(Bagnoli sEMG system, Delsys Inc, Natick, MA, USA) with 5 surface pin-sensors (0.5 mm diameter) was used to record activities of the FDI muscle. The pin-sensors were placed at the center and at the corners of a 5 ×5 mm square. Protruding from the housing of the electrode, the pins of such sensors have blunted ends and do not puncture the skin when pressed against it to detect muscle activities. The analog EMG signals from each pin-sensor were amplified (gain = 1000) and filtered with a bandwidth of 20–450 Hz [24]. A high sampling rate of 20 KHz was used to avoid introducing phase skew across channels for this decomposition-based surface EMG system [24, 25, 26]. Multi-channel surface EMG signals were derived from pair-wise subtractions of voltages (4 single differential EMG channels) at the five pin-detections. This type of spatial filtering allows accurate decomposition of the single motor unit action potential (MUAP) [24, 25, 27], as baseline noise is strictly controlled to a peak-to-peak value of less than 20 μV. The software used for the post-decomposition processing of action potential morphology was EMG works v.4.1 (Delsys Inc, Natick, MA, USA), which can extract action potential “templates” of as many motor unit action potential trains (MUAPTs) as practically possible using an enhanced artificial intelligence algorithm, according to a previous proof-of-principle [25, 26]. The validity of the EMG decomposition of each MUAPT was evaluated with the Decomposition-Synthesis-Decomposition-Compare (DSDC) test [25, 28], the algorithm of the Bagnoli sEMG system. The mean decomposition accuracy was obtained by averaging the individual accuracies of all of the MUAPTs of experimental trials of the feedback condition.

### Data Analysis

To remove involuntary tremulous movements independent of voluntary error corrections, the force signal of the index abduction was first conditioned with a low-pass filter (cut-off frequency: 6 Hz) [7,29]. The remaining force components under 4 Hz are susceptible to most of the effects of visuo-motor processes [3, 6]. In this study, we did not account for high-frequency tremulous force movements (such as 8–12 Hz physiological tremor), as they are functionally irrelevant to correcting force deviations in a visuomotor task [7, 30, 31]. After deleting the first and last 7 seconds of force tracing, force data in the time window of interest were further analyzed (Fig 1). Force fluctuations, obtained following removal of a linear trend from the real force output, were down-sampled at 100 Hz prior to feature extraction [8]. Temporal features of force fluctuations were characterized with the root mean square (RMS) and sample entropy (SampEn). SampEn is a popular and reliable entropy measure of the temporal aspects of biological variability [32]. The mathematical formula for sample entropy was

$$\text{S}ampEn\left(m,r,N\right)=\text{ln}\left(\frac{\sum_{i=1}^{N-m}n_{i}^{m}}{\sum_{i=1}^{N-m-1}n_{i}^{m+1}}\right)=\text{ln}\left(\frac{n_{n}}{n_{d}}\right)$$

where *r* = 15% of the standard deviation of the force channel and *m* = 3. A larger value represents a more complex structure of the low-frequency force fluctuations, and vice versa. Spectral dimensions of force fluctuations were characterized with the mean frequency (MF), spectral dispersion (P_disp_), and ratio of high frequency components (R_HF_) of force fluctuations. The MF was determined through a force spectral profile estimated with a fast Fourier transform and the Welch method (Hanning window, window length: 2.048 seconds, overlapping time segment: 1/4 × window length) with a spectral resolution of 0.1 Hz. Spectral dispersion of force fluctuations was defined as the spectral ranges that consisted of 90% of the overall spectral areas between the lowest 5% and the highest 5% of the spectral regions. The R_HF_ was the ratio of the spectral area above 1.5 Hz to the overall spectral regions of the force fluctuations.

**Fig 3:**
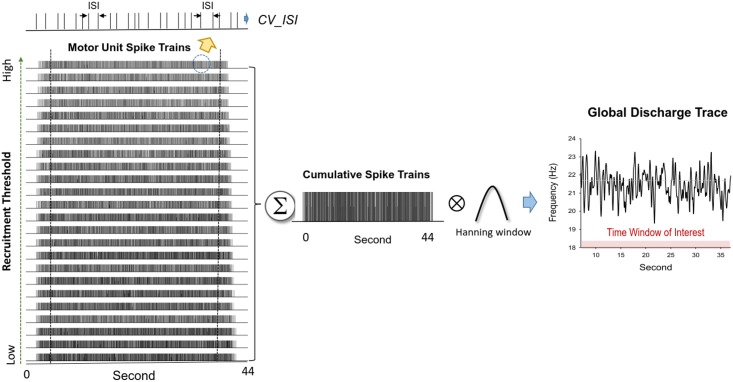
Decomposition of motor unit discharge from multi-channel surface EMG and discharge patterns of individual motor units. Means (Mean ISI) and coefficients of variance (CV_ISI) of inter-spike intervals of individual motor units are assessed with a single motor unit action potential train. The spike trains are arranged from the bottom to the top according to recruitment thresholds of motor units. Global discharge trace was defined as convolution of the cumulative spike trains of all motor units with a Hanning window of fixed duration. Discharge-force relation in this study was characterized with either linear or non-linear association between detrended global discharge trace and detrended force fluctuations.

With decomposition processing, binary spike trains that coded the activation of all motor units with a value of 0 or 1 were identified. In line with the truncated force data, the discharge variables were determined in the time window of interest (00:07 to 00:37) based on the decomposed EMG data of overall 44 seconds (Fig 3). The order of MU recruitment could also be identified by inspecting the decomposed spike trains of all MUs during the early ramp-up contraction (00:04 to 00:05). The motor units recruited above 5% MVC (half of target force) were denoted as higher-threshold motor units (HT MUs) in this study. Experimentally observed variability of the inter-spike interval for individual MUs in the time window of interest was characterized with the coefficient of variation (CV). The grand average of ISI CV (ISI CV__GAV_) was the mean of the ISI CV of a population of motor units. The mean inter-spike interval and ISI__GAV_ of all MUs and HT MUs were determined, respectively.

For each experimental trial, the degree of correlation between spike trains for all MUs and HT MUs was calculated in the frequency domain, known as the pooled coherences MUs of composite spike trains. Prior to calculation of the pooled coherences MUs, the two groups of MUs from all identified MUs or from HT MUs were predetermined. Then the coherence was analyzed with two cumulative spike trains, obtained by summing five motor unit spike trains in each defined MU group (two MU groups composed of all identified MUs or two MU groups simply composed of the HT MUs). This number of MU spike trains was used to derive the cumulative spike trains as an empirical trade-off between the minimum number of motor units and a high coherence sensitivity with a sufficient number of MUs [33]. For an experimental trial of the three feedback conditions, the coherence obtained values were averaged across 200 combinations of different cumulative spike trains. It has been demonstrated that the correlation between cumulative trains of discharges of motor units can enhance low-frequency common oscillatory inputs to motor units relative to traditional coherence estimation by computing the coherence between pairs of motor units, due to effective suppression of individual synaptic inputs to motor units [12, 34]. The magnitude squared coherence values (C) were estimated with two unfiltered composite spike trains using a 1-s Hanning window and an overlap of 90% [35]. The coherence values were converted to Fisher's values (FZ) to minimize an intrinsic bias of the coherence estimates from each segment profile [36]:

$$FZ=tan^{-1}\left(\sqrt{C}\right)$$

Following the z transformation, we subtracted the mean coherence between 100 and 500 Hz from each coherence profile because the mean coherence contained no significant coherence [14,37]. Typically, the corrected pooled MU coherence contained two prominent spectral peaks in the range of 0–4 Hz (ZC_0-4Hz_) and 13–35 Hz (ZC_13-35Hz_). Based on all MUs or those HT MUs, ZC_0-4Hz_ and ZC_13-35Hz_ of the corrected pooled MU coherence were determined subject-by-subject for each experimental trial. Those coherence variables of the three trials were averaged and compared across various error feedback conditions. Differences in the ZC_0-4Hz_ and ZC_13-35Hz_ represent differences in the strength of common oscillatory input to MUs in each band.

Associations between force fluctuations and discharge patterns of motor units were denoted as the force-discharge relation in this study. Prior to assessing the force-discharge relation, we first estimated the cumulative spike trains of all identified MUs followed by a smoothing procedure with Hanning windows of different durations (50 ms, 400 ms, and 1600 ms), with which the degree of tremulous discharges was differentially suppressed [38]. The resulting low-frequency global discharge trace, or effective neural drive to muscle, has been shown to reliably predict force fluctuations during static isometric contraction [34]. In addition to cross correlation, the association between detrended force fluctuation and detrended global discharge trace was characterized with cross mutual information (MI) and cross sample entropy (cross-SampEn), on account of the potential nonlinear effects of summing motor-unit potentials to reproduce the patterns of force fluctuations [39]. Based on the joint probability distribution, MI was used to measure the information shared by force fluctuations and global discharge trace. MI is mathematically formulated as:

$$MI(X;Y)=\sum_{y\in Y}\sum_{x\in X}p(x,y)\text{log}\left({p(x,y)/p}_{1}(x)p_{2}(y)\right)$$

where *p*(*x*,*y*) is the joint probability density function of the time series of *X* and *Y*, and *p*_1_(*x*) and *p*_2_(*y*) are the marginal probability density functions of *X* and *Y*, respectively. With formulas suggested to estimate the Kolmogorov entropy of a time series, cross-SampEn quantifies asynchrony between two distinct but interactive variable statistics [32]. The simplified mathematical expression is

$$Cross-SampEn(m,r,N)=-\text{ln}(\frac{A^{m}(r)(X|Y)}{B^{m}(r)(X|Y)})$$

We applied cross-sample entropy with m = 3 and r = 0.15 (where m is the embedding dimension and r is the tolerance limits of similarity) to a standardized time series of detrended global discharge trace and force fluctuations, and therefore the complexity measure was independent of magnitude of the two analyzed signals. Smaller values of cross-SampEn showed higher coupling of the global discharge trace and force fluctuations. All of the force variables and discharge variables of the three experimental trials were calculated subject-by-subject and then averaged across subjects in different feedback conditions.

### Statistical Analysis

The major interest of this study was to examine the effect of manipulating the size of error feedback via visual feedback on low-frequency force fluctuations, discharge behaviors of MUs, and force-discharge relation. We used repeated measures one-way ANOVA to contrast the force fluctuation variables (RMS, SampEn, MF, spectral dispersion, and ratio of high frequency components), decomposition variables (mean number of decomposed MUs and mean decomposition accuracy), discharge variables (ISI__GAV_ and ISI CV__GAV_), pooled MU coherence variables (ZC_0-4Hz_ and ZC1_3-35Hz_), and discharge-force relationship (cross correlation, MI, and cross-SampEn) among the LAF, NAF, and HAF conditions. The level of significance was 0.05. Signal processing and statistical analyses were completed using Matlab (Mathworks Inc., USA) and the statistical package for Social Sciences (SPSS) for Windows v. 15.0 (SPSS Inc., USA). Data reported in the texts and figures without specific illustrations are presented as mean ± standard error.

## Results

### Force Fluctuation Characteristics

**Fig 4:**
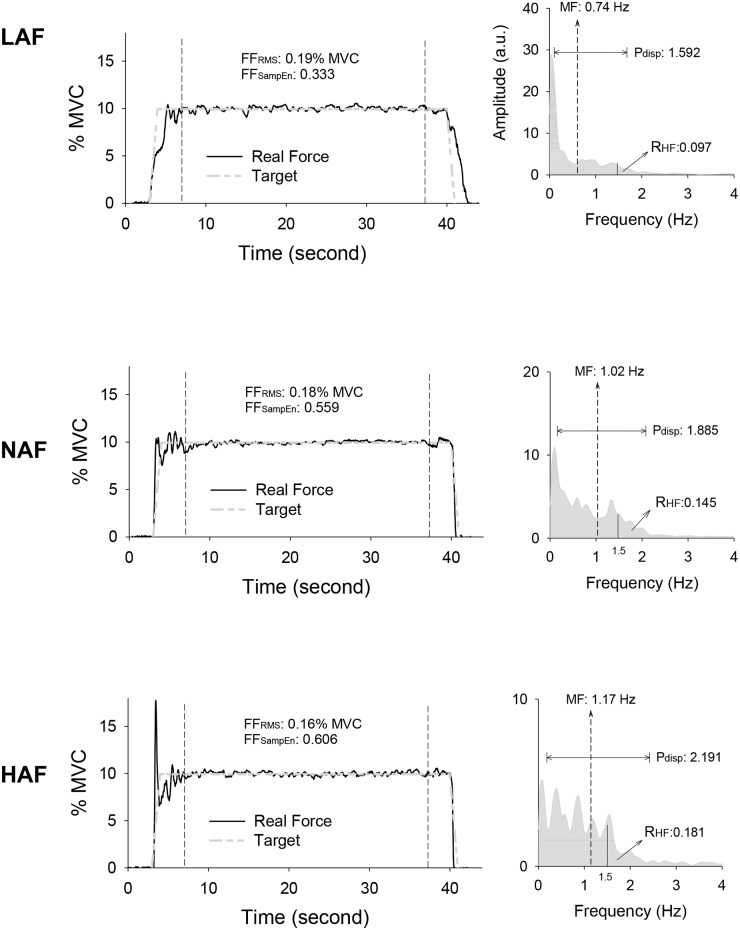
Representative trials of force data (left plots) and pooled power spectra (right plots) of force fluctuations in the time window of interest from a typical participant for the low amplification (LAF), normal amplification (NAF), and high amplification (HAF) conditions. FF_RMS_: root mean square value of force fluctuations, FF_SampEn_: Sample entropy of force fluctuations, MF: mean frequency, P_disp_: power dispersion of force fluctuations, R_HF_: ratio of high frequency components in force fluctuations.

**Fig 5:**
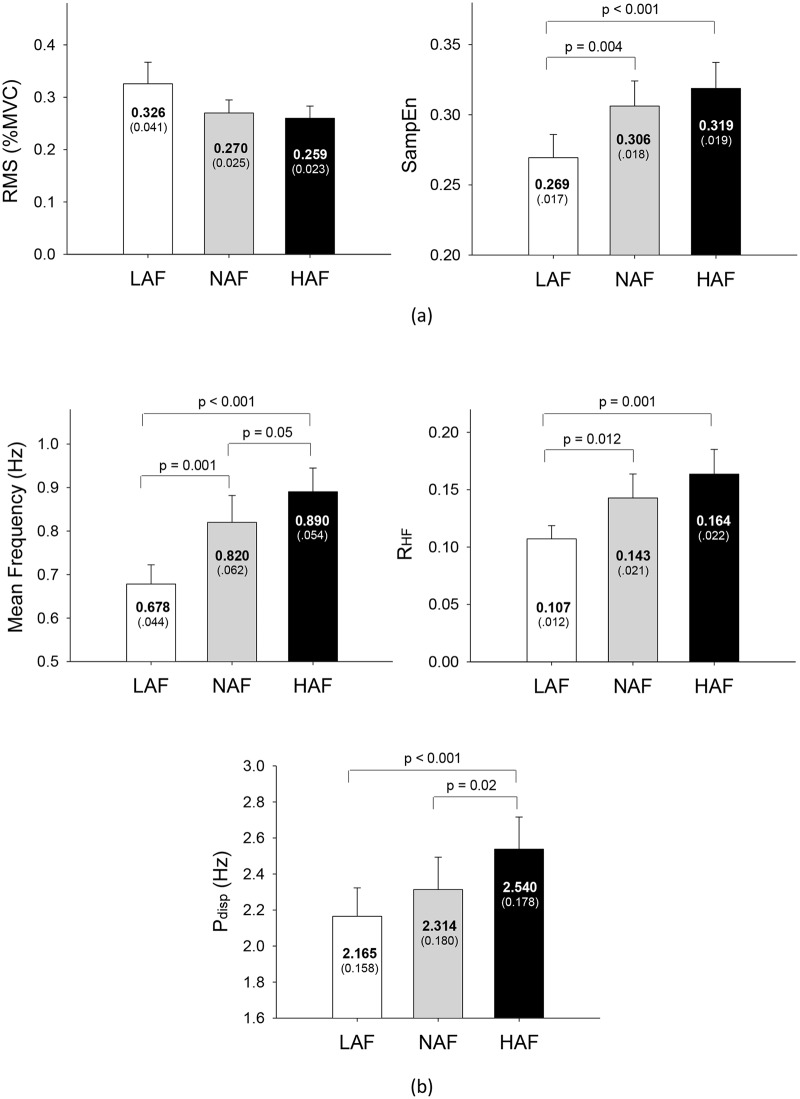
The contrast of force fluctuation variables among the low amplification (LAF), normal amplification (NAF), and high amplification (HAF) conditions. (a) Temporal features of force fluctuations, including RMS and sample entropy (SampEn). (b) Spectral features of force fluctuations, including mean frequency, P_disp_: power dispersion of force fluctuations, R_HF_: ratio of high frequency components in force fluctuations of force fluctuations.

The mean value of MVC of all 14 subjects was 24.07 ± 8.12 N. Fig 4 displays the force output and target signal of a representative trial and the pooled spectra of force fluctuations of three trials from a typical subject for different feedback conditions. In terms of % MVC, Fig 5(a) contrasts the temporal features (RMS and SampEn) of force fluctuations among different error feedback conditions. The repeated measures ANOVA test revealed only a marginal effect of error feedback on the size of the force fluctuations (F_2,26_ = 3.02, *p* = 0.066), with a decreasing trend of smaller force fluctuations for increasing size of error feedback. The complexity of force fluctuations in terms of SampEn was subject to manipulation of error size (F_2,26_ = 13.02, *p* < 0.001)(Fig 5(a)). Reduction in error feedback led to the smallest SampEn of force fluctuations among the feedback conditions (LAF < HAF, NAF, *p* < 0.005). For the spectral dimension, the mean frequency (MF) and ratio of high frequency components of force fluctuations (R_HF_) were also a function of error amplification factor (mean frequency: F_2,26_ = 24.85, *p* < 0.001; R_HF_: F_2,26_ = 8.85, *p* = 0.002). The MF increased progressively with increment of error feedback gain (HAF > NAF > LAF, *p* ≤ .05)(Fig 5(b)), and R_HF_ was lower in the LAF condition than in the NAF and HAF conditions (*p* < 0.05). The ANOVA test also revealed that power dispersion of force fluctuations (P_disp_) was a function of error amplification factor (F_2,26_ = 10.44, *p* < 0.001), and the HAF presented the largest P_disp_ of the three conditions (*p* < 0.05). The findings strongly suggest that temporal and spectral dimensions of force fluctuations varied with the visualized error size during constant isometric contraction.

### Discharge Characteristics and Common Oscillatory Inputs

The average number of motor units in an experimental trial following the decomposition procedure did not differ among the three feedback conditions (F_2,26_ = 0.28, *p* = 0.761)(LAF: 28.1 ± 1.4 (18.0–36.7); NAF: 28.1 ± 1.3 (19.7–38.0); HAF: 28.9 ± 1.4 (16.4–34.7)). The overall decomposition accuracy of motor units was not different among the feedback conditions (F_2,26_ = 0.91, *p* = 0.414)(LAF: 95.22 ± 0.23% (92.82–96.03%); NAF: 94.88 ± 0.30% (92.10–96.38%); HAF: 95.24 ± 0.28% (range: 92.37–96.53%)). Table 1 summarizes the error feedback effect on ISI__GAV_ and ISI CV__GAV_ for all motor units and higher-threshold motor units (HT MUs) among the different feedback conditions. For all motor units, the ANOVA test revealed an insignificant effect of error size on ISI__GAV_ and ISI CV__GAV_. For the HT MUs, ISI CV__GAV_ significantly depended on error amplification factor (F_2,26_ = 4.03, *p* = 0.030), with the highest variations in inter-spike intervals for the HAF conditions (*p* = 0.039). However, ISI__GAV_ was independent of error amplification factor.

**Table 1:** Mean and standard errors of inter-spike interval variables of all motor units and motor units of high threshold in the LAF, NAF, and HAF conditions.

		LAF	NAF	HAF	Statistic
**All Mus**	ISI__GAV_ (ms)	51.99 ± 2.72	52.73 ± 3.16	54.01 ± 3.14	F_2,26_ = 1.01, p = 0.379
	ISI CV__GAV_	0.216 ± 0.006	0.218 ± 0.006	0.224 ± 0.007	F_2,26_ = 2.48, p = 0.104
**HT Mus**	ISI__GAV_ (ms)	57.70 ± 3.03	58.96 ± 3.60	59.66 ± 3.51	F_2,26_ = 0.88, p = 0.425
	ISI CV__GAV_	0.224 ± 0.007	0.229 ± 0.008	0.237 ± 0.009*	F_2,26_ = 4.03, p = 0.030

LAF, low amplification factor; NAF, normal amplification factor; HAF, high amplification factor; All Mus, all motor units; HT Mus, higher-threshold motor units.

*: HAF > NAF, LAF, p = 0.039

**Fig 6:**
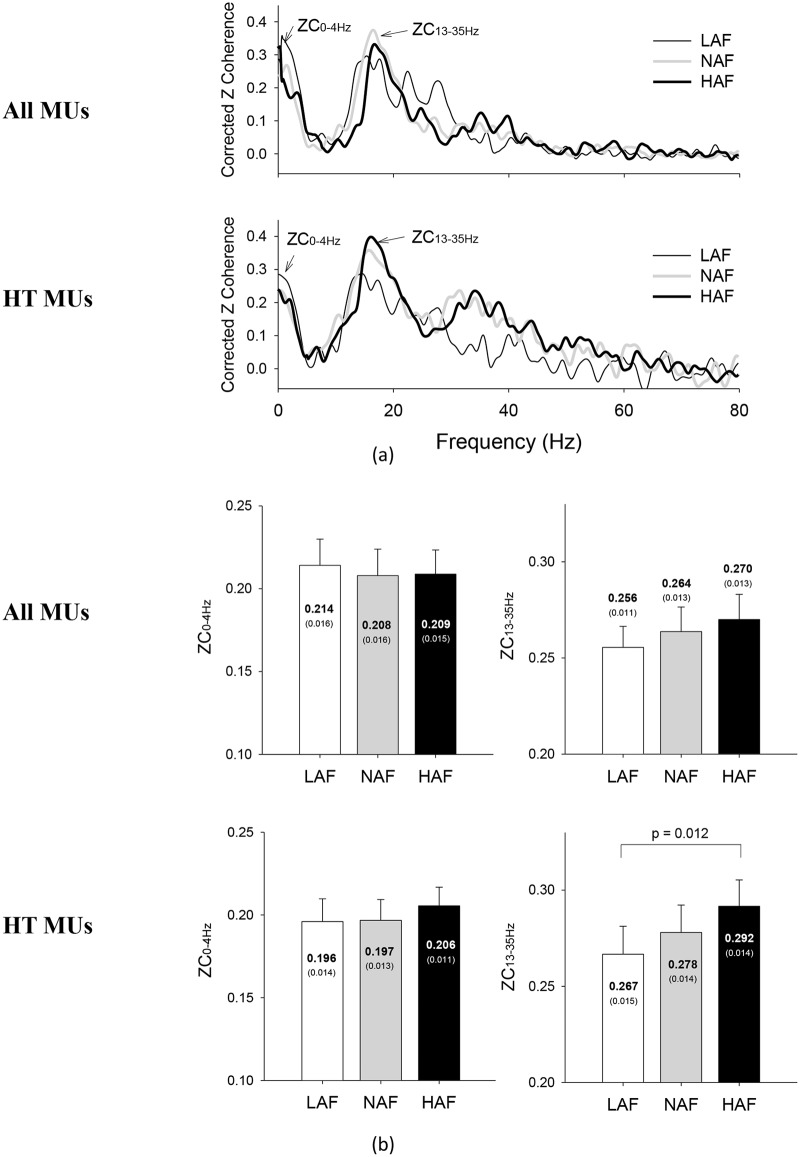
The contrast of pooled motor unit coherence among the low amplification (LAF), normal amplification (NAF), and high amplification (HAF) conditions. (a) Pooled motor unit coherence following z transformation and baseline correction from a typical subject. The pooled motor unit coherences for all motor units and motor units of high threshold contain two major spectral peaks in 0–4 Hz (ZC_0-4Hz_) and 13–35 Hz (ZC_13-35Hz_). (b) Mean and standard errors ZC_0-4Hz_ and ZC_13-35Hz_ of all motor units and motor units with high threshold among the three different feedback conditions. (HT MUs: high-threshold motor units)

Fig 6(a) shows representative pooled motor unit coherences estimated based on all MUs and those HT MUs under various feedback conditions, respectively, from a typical participant. Each pooled motor unit coherence showed two major coherence peaks at 0–4 Hz (ZC_0-4Hz_) and 13–35 Hz (ZC_13-35Hz_). The results of ANOVA showed that the ZC_0-4Hz_ of all MUs (F_2,26_ = 0.28, *p* = 0.760) and HT MUs (F_2,26_ = 0.74, *p* = 0.488) was not significantly affected by variations in error amplification factor. In contrast, ZC_13-35Hz_ of HT MUs was a function of error amplification factor (F_2,26_ = 5.15, *p* = 0.013), although the size effect was not evident for ZC_13-35Hz_ of all MUs (F_2,26_ = 1.83, *p* = 0.181). Increases in error amplification factor were associated with an increasing trend of ZC_13-35Hz_, which was significantly larger in the HAF condition than in the LAF condition (*p* = 0.012).

### Force-discharge Relation

**Fig 7:**
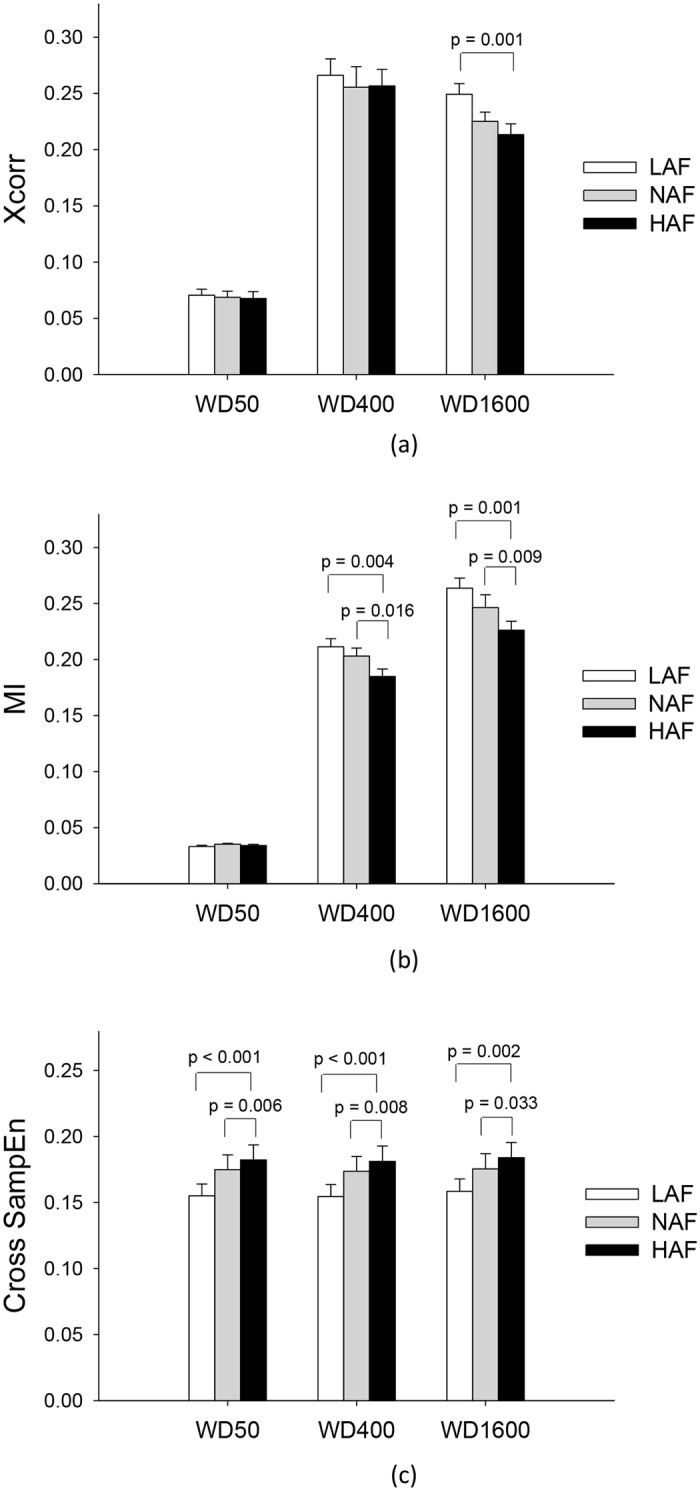
The contrast of force-discharge relations among the three different feedback conditions. The force-discharge relations were characterized as cross correlation (a), mutual information (b), and cross sample entropy (c) under various Hanning window durations (WD) used to smoothen the discharge rate. (Xcorr: cross correlation; MI: mutual information; Cross-SampEn: cross sample entropy)(WD50: window duration 50 ms; WD400: window duration 400 ms; WD1600: window duration 1600 ms).

Fig 7(a)–7(c) summarize the results of the effect of visualized error size on the association of force fluctuations and global discharge trace, characterized with cross correlation, mutual information, and cross sample entropy. The size effect of force-discharge relation characterized with cross-correlation was significant only when an extremely long Hanning window duration (1600 ms) was used to smoothen the global discharge trace (window duration 50 ms: F_2,26_ = 0.11, *p* = 0.892; window duration 400 ms: F_2,26_ = 1.88, *p* = 0.173; window duration 1600 ms: F_2,26_ = 10.26, *p* = 0.001). The use of mutual information to characterize force-discharge could show a significant effect of visualized error size in the window durations of 400 ms (F_2,26_ = 8.78, *p* = 0.001) and 1600 ms (F_2,26_ = 10.75, *p* < 0.001). The relation of force fluctuations and global discharge trace decreased with increasing error feedback gain (*p* < 0.05). The use of cross sample entropy to characterize the force-discharge relation resulted in a consistent of size effect for all window durations (window duration 50 ms: F_2,26_ = 5.43, *p* = 0.011; window duration 400 ms: F_2,26_ = 9.22, *p* = 0.001; window duration 1600 ms: F_2,26_ = 6.00, *p* = 0.007). There was a progressive increase in the degree of asynchrony between force fluctuations and global discharge trace with increasing error amplification factor (*p* < 0.05).

## Discussion

The novel findings of this study were that 1) error-augmentation feedback led to more high-frequency components, power dispersion, and irregularity of low-frequency force fluctuations than did error-reducing feedback, 2) error-augmentation feedback increased the standardized variability and pooled motor unit coherence at 13–35 Hz of the high-threshold MUs relative to those of error-reducing feedback, and 3) non-linear approaches were better able to characterize the size effect on the association of force fluctuations and global MU discharge. The force-discharge relation waned with increasing size of error feedback.

### Behavioral Mechanisms Underlying the Effect of Error Feedback Size

As low-frequency fluctuations are functionally linked to the voluntary engagement of error corrections with the sampled process [30, 31, 40, 41], the reduction in R_HF_ and mean frequency of force fluctuations (Fig 5(b)) jointly suggested a more sporadic error correction to remedy force deviation from an ideal trajectory in the LAF condition. Furthermore, force fluctuations of lower SampEn in the LAF condition indicated coarse-grained force gradation during force-tracking with visual feedback of smaller error size. Hence, it is very likely that virtual reduction in the size of execution error dampened some useful error information and oversimplified control of the force-tracking act in the LAF condition. The low complexity of force fluctuations was reminiscent of the loss of physiological complexity during exhaustive contraction [29] and aging processes [42, 43]. In contrast, the parametric changes in force fluctuations in the HAF condition implied more explorations toward target exactness, which conceptually support rapid motor adaptation with error-augmentation feedback from observations of point-to-point movement with visuomotor rotation [17, 18, 19].

### The Effect of Error Feedback Size on Motor Unit Control

To the best of our knowledge, no study has directly assessed how MU behaviors are tuned to the size of error feedback on a visual display during static isometric contraction. Error-augmentation feedback during static force-matching at 10% MVC led to a significant increase in the standardized variability of the inter-spike intervals (ISI CV__GAV_) of the high-threshold MUs as compared to the NAF and LAF conditions (Table 1). Our observations are consistent with a parallel effect of discharge variability and force fluctuations during low-level static contraction [34, 44]. More specifically, the effect of manipulation of error amplification factor on discharge variability was ascribed to higher-threshold MUs, which generally exhibited more variant discharge with a lower discharge rate and larger twitch force than lower-threshold MUs. Hence, it is of interest to specify common neuromodulatory inputs to populations of higher-threshold MUs. Another potential source of increases in the variability of inter-spike intervals is enhanced synaptic noises or variations in post-spike after-hyperpolarization [45], which have relatively little influence on the control of force steadiness due to an effective cancellation effect in the motoneuronal pool [12,34].

Common input components were enhanced in pooled MU coherence following a summation of multiple spike trains [12, 34, 46]. Previous studies have shown that the delivery of low-frequency common oscillatory inputs (< 4 Hz) to motor neurons is a key determinant of accuracy in force control [12, 47, 48]. The amplitude of 0–4 Hz common oscillatory inputs has been shown to be negatively related to force steadiness [49], and the greater amplitude of 0–4 Hz common oscillatory inputs is associated with the larger size of force fluctuations [34]. In this study, the insignificant modulation of the magnitude of force fluctuations (Fig 5(a)) was compatible with the observation that 0–4 Hz common oscillatory inputs did not differ with feedback condition (Fig 6). The size-dependent modulation of spectral and complexity features of force fluctuations was seemingly contingent upon 13–35 Hz pooled motor unit coherence (Fig 6(b)), though its role on force control was much less addressed. Pooled MU coherence in the β-band can be of central origin and be related to interaction between cortical and spinal circuits [12, 34, 46]. Due to an increasing demand for sensorimotor integration, the β-band coherence showed an increasing trend with increasing effort [35] and a greater amount of spatial visual information of a force task [14]. In fact, enhanced EEG-EMG coherence in the β band is critical to maintaining steady-state force control with precision [50, 51, 52]. Perez et al [53] reported that a marked increase in EEG—EMG coherence around 15–35 Hz was observed during a training session of visuomotor tracking [53], as a consequence of the higher degree of attention and precision control required for early consolidation of learning a novel force task. If the 13–35 Hz of pooled MU coherence is really a corollary to EMG-EEG in the β-band, the error-augmentation feedback in this study could serve to facilitate force-tuning and better exploit visual attention on force-tracking trajectory. Therefore, the potentiation of β-band pooled MU coherence in the HAF condition explains concomitant behavioral changes in force fluctuation property that favor a decreasing trend of smaller size of force fluctuations and increases in strategic complexity and frequent force adjustments to remedy force deviations during force-tracking (Fig 5). A notable point is that the modulation of force gradation using error-augmentation feedback was selective, for only high-threshold MUs were subject to size-dependent modulation of the β-band common oscillatory inputs (Fig 6(b)). Similarly, selective control of lower-threshold and higher-threshold MUs was noted during fatiguing contraction [54] and fast oscillatory contraction [24].

It should be emphasized that the neurophysiological mechanism underlying the size manipulation of error feedback was fundamentally different from that of manipulation of the amount of visual gain (or the number of pixels to represent force change) during static force production [14]. The reason was apparent, since the visual gain was identical in the three feedback conditions in this study. In actual practice, if visual gain of a force task decreases, changes in visual angle with respect to a fixed degree of force fluctuations are smaller in the low-visual gain condition. Visual angle is known to be the most important informational variables of task accuracy for a force task [55]. Several previous experiments on isometric force control showed that a low visual gain condition often associated with one-tenth of the number of visual angle in a high visual gain condition [55]. A smaller visual angle hampers detection of the spatio-temporal information from the movement and its feedback in visual space. The experimental context adds difficulty to map movement trajectory to feedback positions, resulting in a lower level of irregularity but a larger size of force fluctuations under the condition of low visual gain for healthy adults [7, 8, 9]. As visual gain of all the feedback conditions was identical, we were able to physically manipulate the size of execution error without drastically altering visual angle for all feedback conditions in this study. Hence, neither visual angle nor visual gain could adequately account for differential performance variability and motor unit behaviors, when different error amplification factors were used. Theoretically, manipulation of the size of execution error altered the representation of the predicted “correct response”, and the mismatch response is monitored by frontal executive function [56, 57]. In contrast, visual gain determines visual acuity and somatotopic organizations of a force task. The manipulation of visual gain should alter visuomotor processing principally involving in the visual cortex, premotor cortex, and the right inferior parietal lobule [20, 21]. In fact, a high visual gain during static force-tracking sometimes results in a greater size of force fluctuations [58, 59], together with lower 0–5 Hz and higher 6–12 Hz pooled MU coherence [14]. The discrepancies in force characteristics and pooled MU coherence address distinct mechanisms of the two task paradigms by manipulating the error amplification factor and visual gain.

### The Effect of Error Feedback Size on Force-discharge Relation

Theoretically, the summated discharge time series of all MUs should have accounted for the majority of variability of the force exerted by the muscle. But, force-discharge relation is complicated by viscous resistances of the musculotendon system that greatly attenuate the high-frequency force components [37, 46]. Farina and his colleagues propose that the motoneuronal pool serves as a linear filter to extract low-frequency common inputs to MUs with high linear correlation with muscle force [12, 34]. Central to this argument is the fact that the cumulative spike train of all MUs that are low-pass filtered, the effective neural drive [12, 34], can well represent low-frequency force fluctuations. It is interesting to find the progressive decrease in neural representation of force fluctuations with the global discharge pattern with increasing size of error feedback, characterized by cross sample entropy and lower mutual information of the two data series (Fig 7(b) and 7(c). The results of the two non-linear approaches consistently speak for the fact that low-frequency force fluctuations were less predictable by the effective neural drive in the HAF condition. Under this circumstance, the transformation of neural drive into force fluctuations may rely more on unidentified organizational discharge activities from motoneuron subpopulations and/or the independent inputs, which are considered to have a small influence on force genesis in the normal visual condition [12, 34]. As the cross correlation was not a sensitive indicator to feature the size-dependent effect on force-discharge relation (Fig 7(a), the alterations in force-discharge relation by variations in error amplification factor should be non-linear. In part, the non-linearity is a physiological consequence of selective modulation on high-threshold MUs with visualized error size.

### Methodological Issues and Limitations

Several issues and limitations should be noted. First, the activities of individual MUs were obtained from multi-electrode surface EMG in a noninvasive manner following state-of-the-art decomposition techniques. Despite some inconsistencies in the decomposition frameworks of multi-channel surface EMG [60, 61], the validity of the present surface EMG decomposition algorithm proposed by De Luca et al. [25]and Nawab et al [26] has been independently verified by different studies [15, 27, 62] from both physiological and engineering standpoints. Also, to be rigorous, we conducted the experiment at a relatively low exertion level (10% MVC) to minimize MU waveform superimposition and applied the “reconstruct-and-test” procedure [26, 60] to verify the degree of accuracy of the attained identifications. In good agreement with previous studies reporting decomposition accuracies of 92.5% to 97.6% [25, 28], the high decomposition accuracy of this study (92.1%–96.5%) did not vary with feedback conditions. The standard deviation and coefficient of variation of the firing intervals of the FDI in this study were congruent with known physiological characteristics obtained with indwelling EMG [11, 63]. A methodological merit of surface EMG decomposition was that it could account for force control based on more identifiable MUs of different recruitment thresholds than the intramuscular approach, in that force behaviors are tuned to a population of MUs. Secondly, it would be prudent to generalize the present findings to muscle contraction of other forms (such as force-varying, concentric, and eccentric contractions), which may involve different recruitment strategies and rate coding of MUs.

## Conclusions

This study presents a preliminary investigation of behavioral and neurophysiological mechanisms of force regulation when the size of error feedback varies. Capitalizing on characteristic changes in force fluctuations, error-augmentation feedback provides a more frequent and intricate force gradation process, which could increase the effectiveness of motor training by increasing exploration variability. Force control associated with error-augmentation feedback is pertinent to increases in the neurological degree of freedom for the firing patterns of high-threshold MUs that receive β-band common oscillatory inputs and unidentified neural drives.
